# Alfalfa supplementation timing changes the rumen archaeal and fungal community composition and colonization in pre-weaning lambs

**DOI:** 10.3389/fmicb.2024.1380322

**Published:** 2024-05-09

**Authors:** Kenan Li, Haidong Du, Wenliang Guo, Meila Na, Renhua Na

**Affiliations:** College of Animal Science, Inner Mongolia Agricultural University, Hohhot, China

**Keywords:** alfalfa hay, lambs, archaea, fungi, rumen microbiota, age

## Abstract

The establishment of the rumen microbiota plays an important role in the rumen development. However, little is known about the effects of alfalfa supplementation time on rumen microbiota establishment. Here, a total of 42 Hu lambs, seven-day-old, were chosen for the study. After a week of adjustment, six lambs were sacrificed to establish a baseline. The remaining 36 lambs were randomly split into two groups: one receiving alfalfa hay at 14 days (EAF), the other at 42 days (LAF), both groups received milk replacer and starter pellets. Introducing alfalfa at 14 days of age significantly improved total dry matter intake between 28 and 42 days (*p* = 0.04) and average daily gain from both 14 to 28 days (*p* = 0.04) and 28 to 42 days (*p* < 0.01), but this effect disappears from 56 to 70 days (*p* > 0.05). At 42 days, the abundances of *Naganishia*, *Ascochyta*, and *Neosetophoma* in the EAF group were significantly higher (*p* < 0.05) than those in the LAF group (17.8% vs. 3.97, 10.89% vs. 1.77, and 1.27% vs. 0.09%, respectively). At 56 days, the abundances of *Ascochyta*, *Wallemia*, and *Aspergillus* in the EAF group were significantly lower (*p* < 0.05) than in the LAF group (3.53% vs. 16.40, 8.78% vs. 18.89, and 2.14% vs. 4.69%). At 70 days, *Aspergillus* abundance in the EAF group was significantly higher (*p* < 0.05) than in the LAF group (2.69% vs. 0.85%). The LEfSe analysis showed that *Methanobrevibacter_smithii* was the archaeal biomarker at 14 days in both groups. *Methanobrevibacter_sp_AbM4* was enriched at 56 days in the LAF group. Compared to the LAF group, the specific fungal biomarkers in the EAF group included *Sporobolomyces* and *Bullera* at 14 days, *Naganishia, Didymella, Cleistothelebolus,* and *Alloleptosphaeria* at 42 days, *Ascochyta, Neoascochyta*, and *Alfaria* at 70 days. Correlation analysis results showed strong patterns of association both within and between archaea and fungi, which were influenced by alfalfa supplementation time. In summary, alfalfa supplementation at 14 days of age promotes the growth performance of lambs before weaning, and alfalfa supplementation timing significantly affects rumen archaeal and fungal communities and dynamical changes.

## Introduction

For ruminants, the rumen harbors a complex and diverse microbiota, including bacteria, archaea, fungi, and protozoa (Huws et al., 2018). These microorganisms are critical to feed degradation and provide volatile fatty acids, protein, minerals, and vitamins to the host. However, young ruminants have an immature rumen at 2 weeks of life and cannot utilize forages (Xiao et al., 2023). Previous studies have reported that the rumen development process includes the non-rumination phase (from birth to week 3), the transition phase (from week 3 to 8), and the rumination phase (from week 8 onwards) (Jiao et al., 2015; Yáñez-Ruiz et al., 2015). In the non-rumination phase, the diet of newborn ruminants is dominated by milk, which bypasses the rumen and enters directly into the abomasum. Consequently, the effects of milk on rumen microbiome and fermentation function are very limited. During the transition phase (from week 3 to 8), solid feed gradually increases, resulting in increased rumen anatomic development (rumen mass and papillae), functional achievement (fermentation capacity and enzyme activity), and microbial establishment (bacteria, archaea, fungi, and protozoa) (Jiao et al., 2015). These events promote a key process of transition from a functional non-ruminant to a true ruminant that relies on rapidly establishing the rumen microbiota (Dias et al., 2017). Compared with adult ruminants, the early colonization phases of the rumen microbiota are dynamic and more malleable to be influenced by nutritional manipulations in young ruminants (Li et al., 2023a). In neonatal calves, the ruminal bacteria primarily come from their dams, including the maternal milk, saliva, vaginal, and external environments (Yeoman et al., 2018). The Proteobacteria and Firmicutes were the dominant phylum in the neonatal ruminants (Rey et al., 2014; Li et al., 2019a). With the intake of milk gradually increased, Proteobacteria was replaced by Bacteroidetes as the dominant phylum (Rey et al., 2014). Moreover, some other genera that are commonly found in the mature rumen were already established in the rumen, such as *Prevotella* and *Ruminococcus* (Dias et al., 2017; Zhang et al., 2019). As the intake of solid feed increased, Bacteroidetes and Firmicutes became the dominant phylum. At the genus level, some rumen bacteria that can degrade carbohydrates and fiber gradually increase and maintain relatively stable abundances in the rumen, such as *Prevotella*, *Ruminococcus*, and *Treponema* (Rey et al., 2014; Li et al., 2019a; Yin et al., 2021).

In addition to bacteria, methanogenic archaea were found in the rumen shortly after birth (Guzman et al., 2015). Methanogens can use H_2_ to synthesize methane, which is a greenhouse gas that contributes to global warming, and a relevant source of diet energy loss for the host (Knapp et al., 2014). Lyons et al. (2017) found that the short-term addition of linseed oil to lambs’ diets had lasting effects on the rumen microbiome. Given that manipulation of the rumen microbiome in early life may be the best window, identifying factors that affect methanogenic archaea colonization may guide the development of methane mitigation strategies with long-term implications.

Anaerobic rumen fungi are known to play a key role in the degradation of feed fibers due to the production of various biomass-degrading enzymes and the ability of their rhizomorphs to penetrate fiber structural barriers (Huws et al., 2018). Besides, anaerobic fungi are closely related to methanogens, as anaerobic fungi release H_2_ during the process of fermenting carbohydrates, promoting the methanogenic archaea community (Jin et al., 2014). Thus, the method of co-culture of methanogenic archaea and fungi may provide a viable method for the culture and investigation of as-yet unidentified methanogenic archaea. The rumen fungal community varied considerably in lambs during the pre-weaning period, indicating that the fungal community may be affected by the age of the lambs (Zhang et al., 2019). The age-related genera contained *Acremonium*, *Microascus*, *Valsonectria*, *Myrmecridium*, and so on (Yin et al., 2022). However, the factors affecting the colonization and activity of ruminal fungi in the pre-weaning lambs’ rumen remain unclear.

A clear understanding of the factors that influence the establishment of the rumen microbiome in early life is crucial for enhancing animal performance (Li et al., 2023a). At present, there are relatively many studies on the process of early rumen bacterial colonization and its influencing factors (Lyons et al., 2017; Yang et al., 2018; Li et al., 2023a). However, there have been only very limited investigations of the rumen archaeal and fungal community dynamics in lambs, particularly their community changes in response to alfalfa hay supplementation time in pre-weaning lambs. Therefore, we hypothesized that starting alfalfa supplementation at either 14 or 42 d of age can affect the composition of the rumen microbiota community and that the rumen microbial community changes with age in response to the alfalfa supplementation time, leading to improved growth performance. The objective of this study is to investigate the effects of alfalfa hay supplementation at 14 d or 42 d of age on the growth performance and the composition and dynamic changes of rumen archaeal and fungal communities in pre-weaning lambs.

## Materials and methods

### Animals, diets, and experimental design

This study was performed at the Inner Mongolia Jinlai Animal Husbandry Technology Co., LTD (Hohhot, China) from October to December 2022. The experiment and animal procedures were done according to the ‘Laboratory Animal Guideline for Ethical Review of Animal Welfare’ National Standard of the People’s Republic of China (GB/T35892-2018).

**Figure 1:**
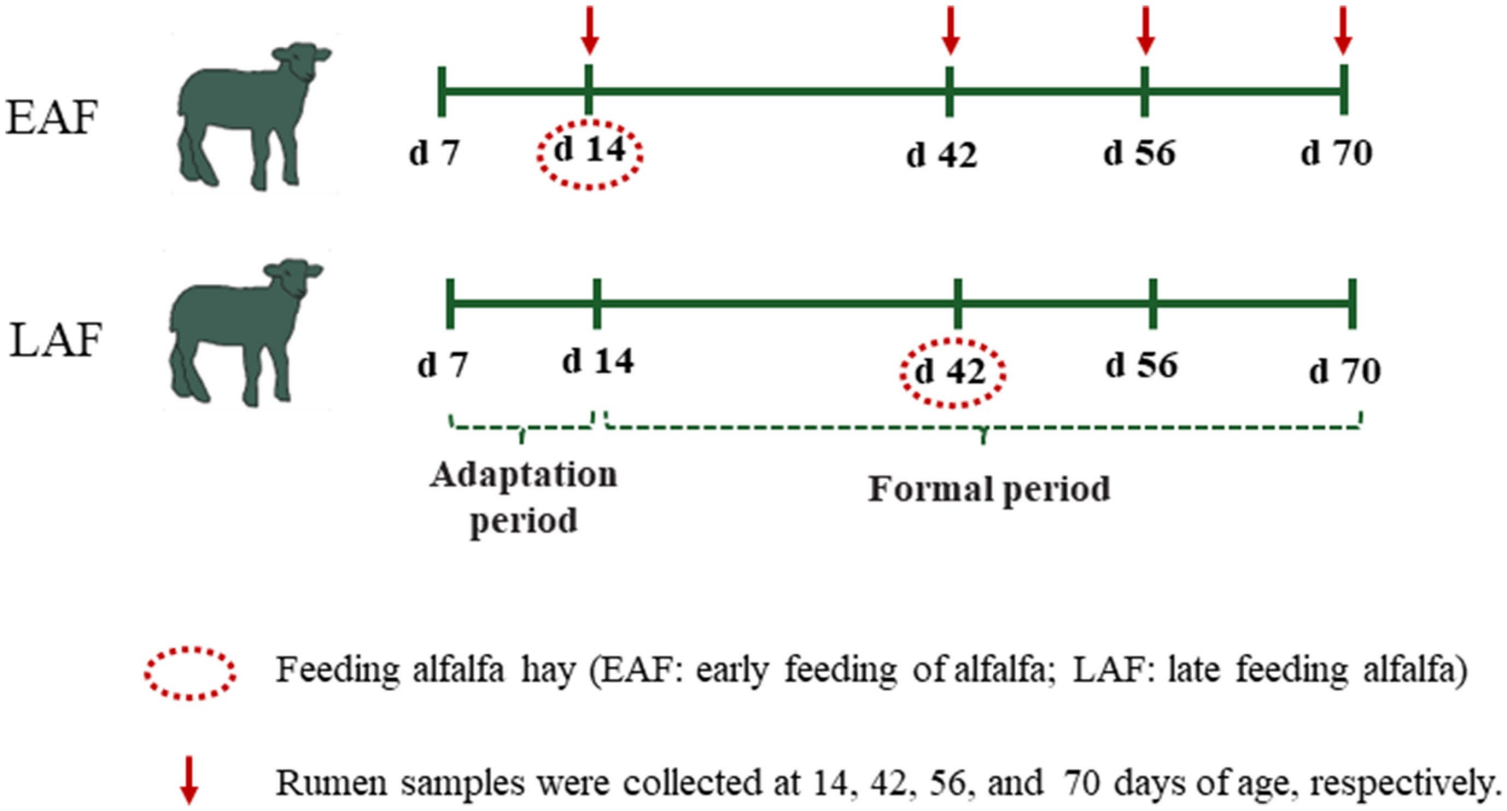
Experimental design and sampling schedule.

This experiment adopted a completely randomized design. A total of 42 male Hu lambs (3.88 ± 0.92 kg) were selected at 7 d of age. After 7 d of adaptation to the environment and milk replacer (MR), six lambs were selected and slaughtered at 14 d of age as a control group (CON). The remaining 36 lambs were fed MR, starter concentrate, and randomly assigned into 2 treatments according to body weight, including alfalfa hay was supplemented at 14 d of age as early alfalfa feeding group (EAF, n = 18); alfalfa hay was supplemented at 42 d of age as late alfalfa feeding group (LAF, n = 18) (Figure 1). There were 6 replicates per treatment and 3 lambs per replicate, each pen houses three lambs. Three buckets were placed inside the hutch, each holding starter concentrate, alfalfa hay, and water, respectively. During the period from 14 to 70 days of age, the feeding amounts of starter pellets and alfalfa hay were recorded daily, while the leftover feed was also collected. The leftover feed was weighed every two weeks. Subsequently, based on the recorded feeding amounts and leftover feed, the intake of starter pellets and alfalfa hay was calculated every two weeks. The body weight (BW) of each lamb was measured on every two weeks basis before the morning feeding during the trial period. The total dry matter intake (total DMI) and average daily gain (ADG) were calculated accordingly. Representative samples of MR, starter pellets, and alfalfa hay were collected and dried at 60°C for 72 h and ground in a roller mill to pass through a 1.0 mm sieve. Then, their chemical compositions, including dry matter (DM, method 930.15), crude protein (CP, method 984.13), ether extract (EE, method 920.39), crude ash (Ash, method 924.05), calcium (method 927.02), and phosphorus (method 984.27) were analyzed following the Association of Official Analytical Chemists (AOAC, 2007) methods. Neutral detergent fiber (NDF) and acid detergent fiber (ADF) were analyzed using the ANKOM fiber analysis equipment (A2000i; ANKOM Technology Corp., Fairport, NY, USA), as described by Van Soest et al. (1991). The ingredients and nutrient composition of the MR, starter pellets, and alfalfa hay are provided in Supplementary Table S1. Alfalfa hay was cut into 2.2 cm using a chaff cutter (9Z-4C, Sida, Luoyang, China). All lambs had free access to concentrate, alfalfa hay, and water. To avoid wasting starter concentrate and alfalfa while the lambs were being fed, we fed less and more frequently.

All lambs were offered MR from 7 to 70 d of age. MR was offered 720 mL/d (based on consumption during the adaptation period) at 15 d of age, then reduced at a rate of 40 mL/d until 200 mL/d. Lambs were offered MR four times a day at 08:00, 12:00, 16:00, and 20:00 h from 7 to 28 d of age, and three times a day at 08:00, 12:00, and 1,800 h from 29 to 70 d of age. The ratio of MR to water was 1:7 (weight(g)/volume (mL)).

Six animals in each treatment (one lamb from each pen) were slaughtered randomly 3 h after the morning feeding at 42, 56, and 70 d of age. The rumen content was collected in triplicates, snap-frozen in liquid nitrogen, and then stored at −80°C for analysis of ruminal microbiota. The slaughterhouse is located in Tumet Left Banner, Hohhot, Inner Mongolia Autonomous Region, China. During transportation, we adhere to the ‘Laboratory Animal Guideline for Ethical Review of Animal Welfare’ National Standard of the People’s Republic of China (GB/T 35892-2018) and strive to minimize transportation time and distance to reduce the stress response of the animals. Additionally, we have professional transportation personnel responsible for supervising the animals to ensure their safety during transportation.

### Bioinformatics and data analysis

Microbial DNA was extracted from the rumen content using the E.Z.N.A. Stool DNA Kit (Omega Biotek, Norcross, GA, United States) following the manufacturer’s instructions. The quality and concentration of the rumen microbial DNA were determined using 1% agarose gel electrophoresis and NanoDrop 2000 Spectrophotometer (Thermo Fisher Scientific Inc., USA). The V4 region of the archaeal 16S rRNA and the fungal internal transcribed spacer (ITS1) were amplified by PCR. The archaeal primers used in the current study were Arch519F: 5’-CAGCCGCCGCGGTAA-3′ and Arch915R: 5’-GTGCTCCCCCGCCAATTCCT-3′. The fungal primers used in the current study were ITS1-1F-F: 5′-CTTGGTCATTTAGAGGAAGTAA-3′ and ITS1-1F-R: 5′-GCTGCGTTCTTCATCGATGC-3′. All PCR reactions were carried out with 15 μL of Phusion® High-Fidelity PCR Master Mix (New England Biolabs); 0.2 μM of forward and reverse primers, and about 10 ng template DNA. The steps of archaeal 16S rRNA and fungal ITS1 genes sequence amplification are as follows: 98°C for 1 min, followed by 30 cycles at 98°C for 10 s, 50°C for 30 s, and elongation at 72°C for 30 s and 72°C for 5 min. The PCR products were detected using 2% agarose gel electrophoresis, and the target fragments were recovered after completion. After the constructed library was quantified by the Qubit and real-time PCR. Sequencing was performed using the Illumina NovaSeq 6,000 sequencing platform.

After original sequencing data was obtained, quality filtering on the raw tags was performed using the fastp (Version 0.23.1) software to obtain high-quality Clean Tags (Bokulich et al., 2012). The tags were compared with the reference database using the UCHIME Algorithm^1^ to detect chimera sequences, and then the chimera sequences were removed (Edgar et al., 2011). Then the effective tags were finally obtained. The effective tags use the DADA2 module in QIIME2 (Version Qiime2-202202) software to eliminate interference and obtain the final amplicon sequence variants. Species annotation was performed using QIIME2 (Version Qiime2-202202) software. For archaeal 16S rRNA, the annotation database is Silva_138.1 database, while for ITS, it is Unite v9.0 database. The absolute abundance of amplicon sequence variants was normalized using a standard sequence number corresponding to the sample with the least sequences. The QIIME2 software (v.1.8.0) was used for statistical analysis of alpha diversity, including Observed_OTUs, Chao 1, and Shannon indices. Wilcox rank sum test was performed to analyze whether a significant difference existed in alpha diversity. Beta diversity on both Bray Curtis and Unweighted Unifrac was calculated by QIIME software. Analysis of similarity (ANOSIM) was used to examine whether a significant difference existed in beta diversity. To investigate the rumen microbiota with significant differences between the EAF group and the LAF group, we used R (Version 3.5.3) software and applied the MetaStat method to test the species abundance data between groups to obtain *p*-values. The software of Linear discriminant analysis Effect Size (LEfSe) (Version 1.0) was used to investigate the effects of age on the dynamic changes of rumen microbiota. Microbial communities having linear discriminant analysis (LDA) score values greater than 3.5 are identified as specific microbiota unique to the treatment group, thereby distinguishing this treatment group from others The rumen archaeal sequencing data and fungal sequencing data of this study are available in the NCBI SRA database with the BioProject ID: PRJNA1067551 and ID: PRJNA1067533, respectively.

Spearman’s rank correlation was used to assess the relationship between the rumen archaea and fungi. Only the top 10 genera that were detected in at least 50% of all samples were included. The *p*-values were adjusted using the Benjamini-Hochberg method. The data were analyzed on the online tool of Majorbio Cloud Platform^2^ (Ren et al., 2022).

### Statistical analysis

The significance of ADG, starter intake, alfalfa hay intake, and total DMI results are analyzed using the independent sample T-test in SPSS (version 26.0) software. The *p*-values less than 0.05 for rumen microbiota, ADG, starter intake, alfalfa hay intake, and total DMI between the EAF and LAF groups at different ages were considered as the criterion for statistical significance, while a trend was considered at *p* < 0.10.

## Results

### Performance

**Figure 2:**
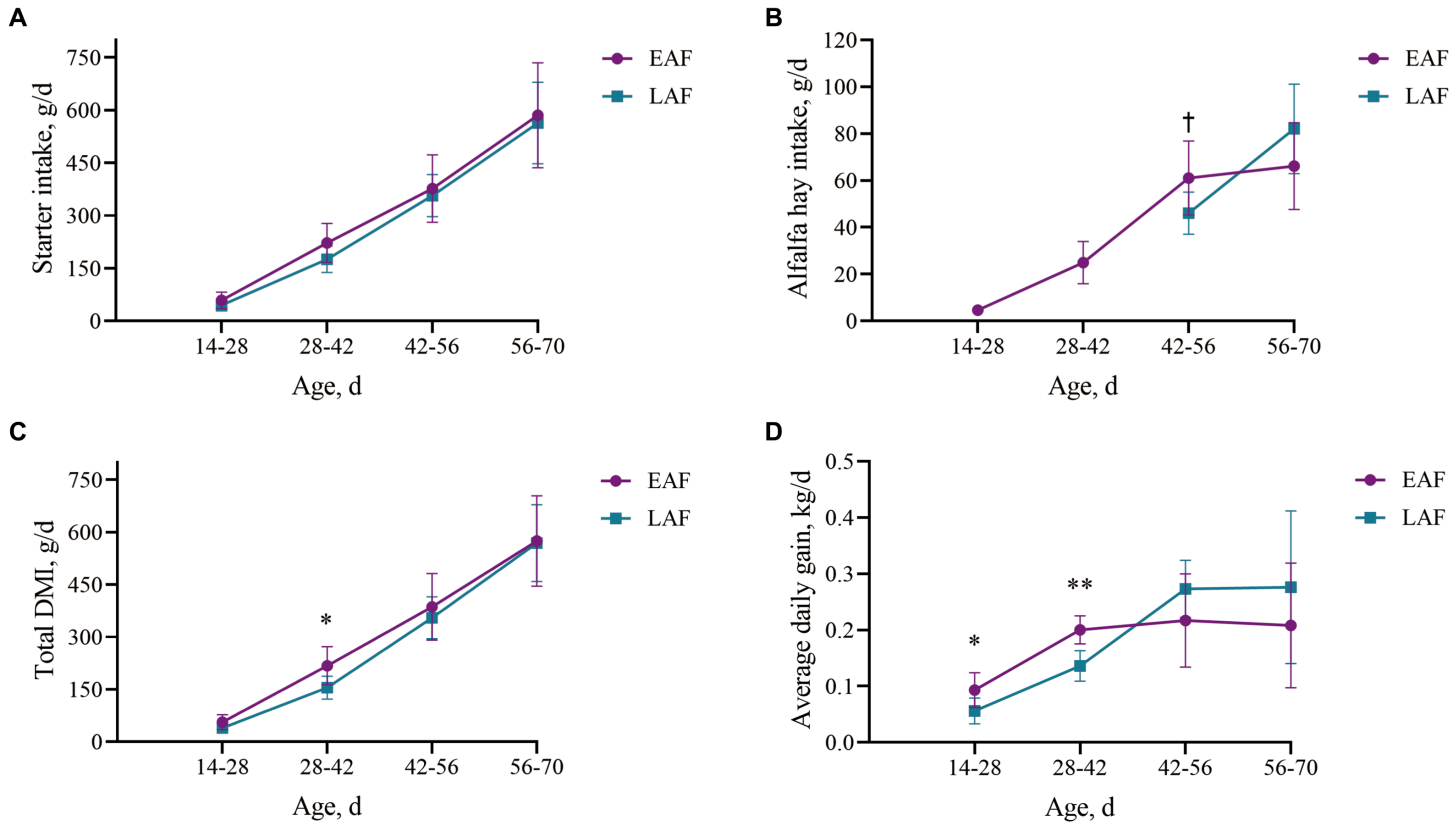
Effect of alfalfa hay supplementation time on performance and feed intake of lambs. The starter intake every two weeks **(A)**, alfalfa hay intake every two weeks **(B)**, total DMI (including starter feed and alfalfa hay) every two weeks **(C)**, average daily gain every two weeks **(D)**. Daggers (†) indicate trends (0.05 < *P* < 0.1) between groups, asterisks (*) indicate differences (*p* < 0.05) between groups, double asterisks (**) indicate differences (*p* < 0.01) between groups. EAF, lambs were fed alfalfa hay at 14 days of age as the early alfalfa feeding group; LAF, lambs were fed alfalfa hay at 42 days of age as the late alfalfa feeding group.

Animal growth performance and feed intake are presented in Figure 2. From 42 to 56 d of age, the intake of alfalfa hay in EAF lambs tended to be higher than that in LAF lambs (*p* = 0.07). The total DMI and ADG at 28 to 42 d of age in the EAF group were significantly higher (*p* < 0.05) than those in the LAF group. In addition, from 14 to 28 d of age, ADG in the EAF group was significantly higher than those in the LAF group (*p* = 0.04).

### The composition of archaeal populations

In this study, after quality filtering, 3,691,359 (mean 87,890 ± SD 3,247 each sample) high-quality archaea sequences were obtained. The Good coverage was greater than 0.99, indicating that our data provide sufficient sequencing depth for the diversity of archaeal communities in all samples.

**Figure 3:**
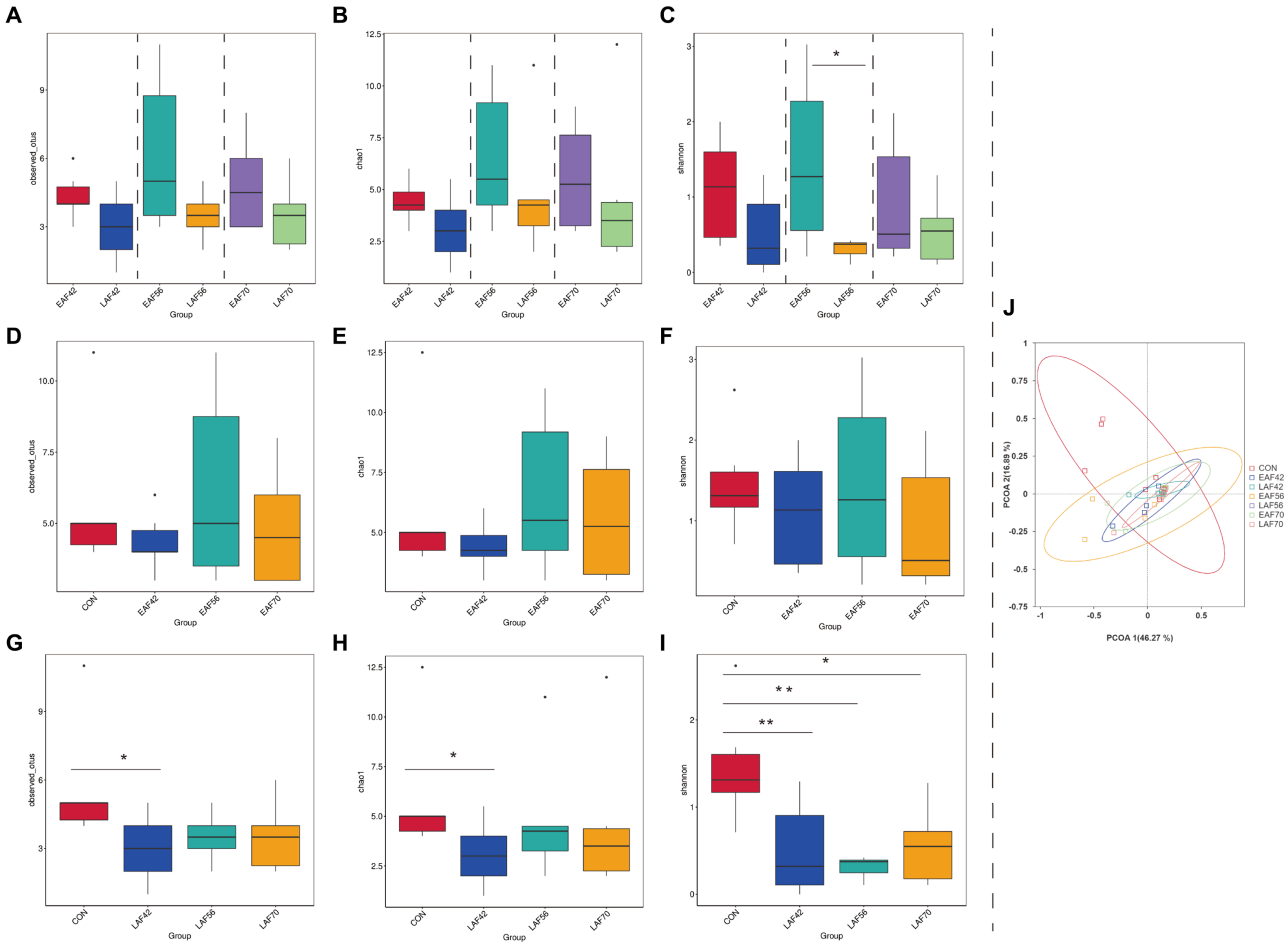
The effect of alfalfa supplementation timing on alpha and beta diversity of rumen archaea in pre-weaning lambs. Observed_OTUs **(A)**, Chao 1 **(B)**, and Shannon **(C)** indices in the rumen archaea between the EAF and LAF groups at 42, 56, and 70 days of age. Alpha diversity of the rumen archaea was analyzed using the Wilcox rank sum test. The rumen archaea diversities of the EAF group at 14, 42, 56, and 70 d of age **(D–F)**, and the alpha diversities of the LAF group at 14, 42, 56, and 70 days of age **(G–I)**. *p < 0.05, **p < 0.01, ***p < 0.001. The principal coordinate analysis (PCoA) was performed based on the Bray-Curtis dissimilarities metric **(J)**. EAF, lambs were fed alfalfa hay at 14 days of age as the early alfalfa feeding group; LAF, lambs were fed alfalfa hay at 42 days of age as the late alfalfa feeding group; CON, lambs were slaughtered at 14 days of age.

For archaeal alpha-diversity analysis, the Observed_OTUs (Figure 3A) and Chao 1 (Figure 3B) indices did not differ by treatment at 42, 56, and 70 d of age, respectively (*p* > 0.05). The Shannon index was higher in the EAF group than in the LAF group at 56 d of age (*p* < 0.05) (Figure 3C). In addition, in the EAF group, the Observed_OTUs (Figure 3D), Chao 1 (Figure 3E), and Shannon (Figure 3F) indices were not affected by age (p > 0.05). However, the Observed_OTUs (Figure 3G) and Chao 1 (Figure 3H) indices were higher in the CON group than in the LAF group at 42 d of age (p < 0.05). The Shannon index (Figure 3I) was higher in the CON group than the LAF group at 42 (*p* < 0.01), 56 (*p* < 0.01), and 70 (*p* < 0.05) d of age. Principal coordinate analysis (PCoA) was performed based on the Bray-Curtis dissimilarities metric (Figure 3J) in the archaeal community. Our beta-diversity analysis showed that Bray-Curtis dissimilarities in the archaeal community between the EAF and LAF groups at 42, 56, and 70 d of age were not significant (ANOSIM, *R* = 0.09, 0.20, −0.10; *p* = 0.09, 0.06, 0.99). For the age effect, the rumen archaea of lambs at 14 d of age were distinct compared to other ages based on Bray-Curtis distance (ANOSIM, *R* = 0.48, *p* = 0.01), whereas variations observed between 42 d vs. 56 d (ANOSIM, *R* = 0.005, *p* = 0.27), or 56 d vs. 70 d (ANOSIM, R = −0.01, *p* = 0.73) were not significant (Figure 3J).

**Figure 4:**
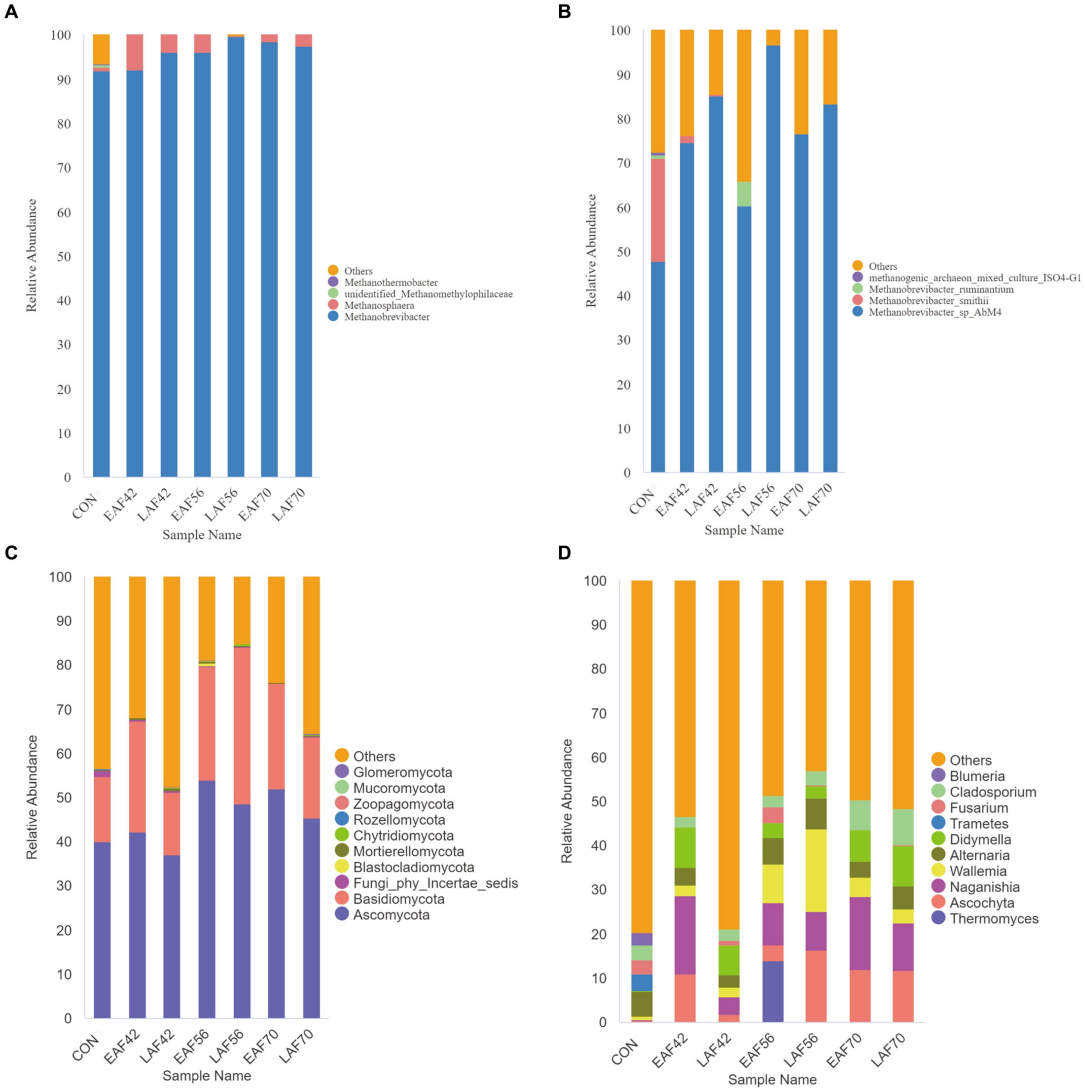
The composition of the rumen archaea and fungi in response to alfalfa supplementation timing. The composition of rumen archaea at genus level **(A)** and species level **(B)**. The composition of rumen fungi at phylum level **(C)** and genus level **(D)**. EAF, lambs were fed alfalfa hay at 14 days of age as the early alfalfa feeding group; LAF, lambs were fed alfalfa hay at 42 days of age as the late alfalfa feeding group; CON, lambs were slaughtered at 14 days of age.

The Euryarchaeota (98.97 ± 4.50%) was the dominant phyla of archaea, including the order *Methanobacteriales* (98.97 ± 4.50%) and *Methanomassiliicoccales* (0.73 ± 0.04%) as well as the genera *Methanobrevibacter* (95.97 ± 6.30%) and *Methanosphaera* (2.94 ± 0.05%) (Figure 4A). At the species level, *Methanobrevibacter_sp_AbM4* (74.90 ± 29.30%) and *Methanobrevibacter_smithii* (3.57 ± 1.34%) dominated the archaeal community (Figure 4B).

**Figure 5:**
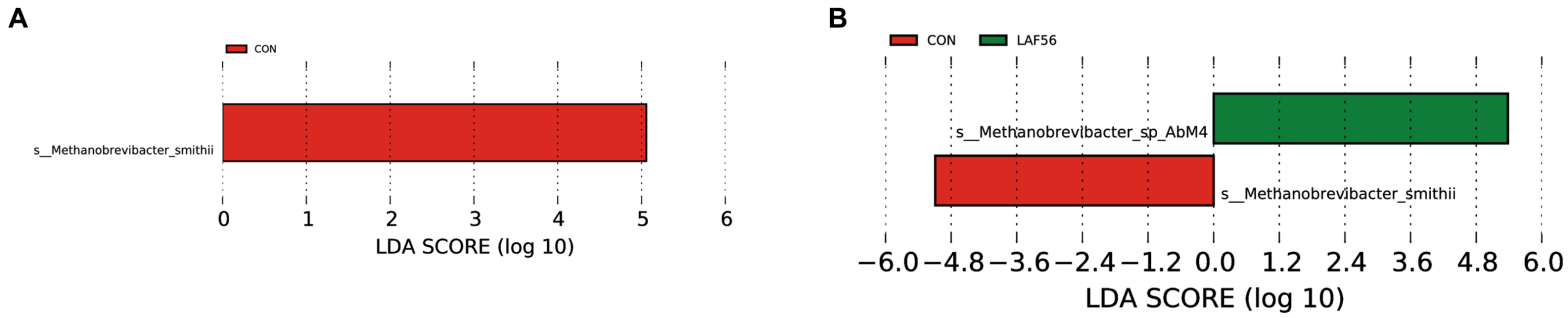
The rumen archaeal biomarker (species level) was identified by LEfSe analysis (LDA Score > 3.5) for each age in the EAF group **(A)** and the LAF group **(B)**. EAF, lambs were fed alfalfa hay at 14 days of age as the early alfalfa feeding group; LAF, lambs were fed alfalfa hay at 42 days of age as the late alfalfa feeding group; CON, lambs were slaughtered at 14 days of age.

A two-way comparison was performed to calculate the differences in archaeal community composition. At 42 and 56 d of age, the abundance of *Methanosphaera* tended to be higher (*p* = 0.05) in the EAF group than in the LAF group (Table 1). However, the abundance of *Methanobrevibacter* was not affected by the treatment at 42 (*p* = 0.09), 56 (*p* = 0.09), and 70 (*p* = 0.31) d of age. Methanogens with LDA scores exceeding 3.5 were deemed to be characteristic of this particular age group, distinguishing it from other ages. In the EAF group, the species *Methanobrevibacter_smithii* was the rumen archaeal biomarkers at 14 d of age (CON group) (Figure 5A). In the LAF group, LEfSe analysis showed that the species *Methanobrevibacter_smithii* was enriched at 14 d of age, and *Methanobrevibacter_sp_AbM4* was enriched at 56 d of age (Figure 5B).

**Table 1:** Differences in the composition of rumen archaea and fungi among different groups (relative abundance above 1.0%).

Item	Day 42		SEM	*p*-value	Day 56		SEM	*p*-value	Day 70		SEM	*p*-value
	EAF	LAF			EAF	LAF			EAF	LAF		
Archaea												
*Methanobrevibacter*	96.06±2.42	99.54±0.29	0.02	0.09	96.06±2.42	99.54±0.29	0.01	0.09	98.38±0.56	97.45±0.83	0.01	0.31
*Methanosphaera*	3.94±2.42	0.23±0.23	0.02	0.05	3.94±2.42	0.23±0.23	0.01	0.05	1.62±0.56	2.55±0.83	0.01	0.22
Fungi												
*Naganishia*	17.80±0.04	3.97±0.04	0.03	<0.01	9.49±0.04	8.61±0.02	0.02	0.94	16.57±0.04	10.85±0.03	0.02	0.17
*Ascochyta*	10.89±0.04	1.77±0.02	0.03	0.01	3.53±0.02	16.40±0.05	0.03	<0.01	12.01±0.02	11.66±0.04	0.02	0.97
*Didymella*	9.22±0.03	6.72±0.04	0.02	0.82	3.48±0.01	2.74±0.01	0.01	0.83	7.13±0.03	9.15±0.03	0.02	0.79
*Alternaria*	3.82±0.01	2.89±0.02	0.01	0.83	6.05±0.05	6.95±0.04	0.03	0.95	3.56±0.01	5.17±0.03	0.01	0.77
*Wallemia*	2.46±0.01	2.14±0.01	0.01	0.89	8.78±0.04	18.89±0.05	0.03	0.03	4.29±0.01	3.23±0.01	0.01	0.75
*Cladosporium*	2.28±0.01	2.59±0.01	0.01	0.94	2.74±0.01	3.22±0.01	0.01	0.91	6.76±0.03	8.12±0.02	0.02	0.83
*Filobasidium*	1.57±0.01	2.67±0.01	0.01	0.66	1.44±0.01	1.71±0.00	0.01	0.93	0.79±0.00	1.84±0.01	0.01	0.17
*Vishniacozyma*	1.56±0.01	0.46±0.00	0.01	0.26	0.40±0.00	0.89±0.00	0.01	0.23	0.58±0.00	0.87±0.00	0.01	0.75
*Neosetophoma*	1.27±0.01	0.09±0.00	0.01	0.01	1.99±0.01	1.10±0.00	0.01	0.79	3.40±0.01	1.56±0.01	0.01	0.21
*Aspergillus*	1.21±0.01	1.95±0.01	0.01	0.78	2.14±0.01	4.69±0.01	0.01	0.04	2.69±0.01	0.85±0.00	0.01	0.04
*Enterocarpus*	1.19±0.01	0.13±0.00	0.01	0.21	0.03±0.00	0.26±0.00	0.01	0.69	0.07±0.00	0.01±0.00	0.01	0.59
*Fusarium*	0.12±0.00	0.88±0.00	0.01	0.03	3.43±0.03	0.09±0.00	0.02	0.22	0.07±0.00	0.16±0.00	0.01	0.56
*Kernia*	0.67±0.00	0.14±0.00	0.01	0.01	1.90±0.02	0.19±0.00	0.01	0.28	0.66±0.00	0.07±0.00	0.01	0.13
*Albifimbria*	0.45±0.00	0.05±0.00	0.01	0.64	1.83±0.01	0.15±0.00	0.01	0.09	0.01±0.00	0.00±0.00	0.01	0.77
*Occultifur*	0.05±0.00	0.08±0.00	0.01	0.84	1.13±0.01	0.26±0.00	0.01	0.75	0.04±0.00	0.04±0.00	0.01	0.98
*Cystobasidium*	0.04±0.00	0.00±0.00	0.01	0.06	1.01±0.01	1.53±0.01	0.01	0.85	0.83±0.01	0.07±0.00	0.01	0.23
*Stagonosporopsis*	0.89±0.00	0.19±0.00	0.01	0.02	0.04±0.00	0.25±0.00	0.01	0.01	1.34±0.01	2.13±0.01	0.01	0.79
*Candida*	0.59±0.00	0.74±0.00	0.01	0.84	0.53±0.00	1.03±0.00	0.01	0.09	1.30±0.01	0.17±0.00	0.01	0.06

EAF, lambs were fed alfalfa hay at 14 days of age as the early alfalfa feeding group; LAF, lambs were fed alfalfa hay at 42 days of age as the late alfalfa feeding group.

### The composition of fungal populations

In this study, after quality filtering, 3,808,742 (90,684 ± 19,486) high-quality fungi sequences were obtained. The Good coverage was greater than 0.99, indicating that our data provide sufficient sequencing depth for the diversity of fungal communities in all samples.

**Figure 6:**
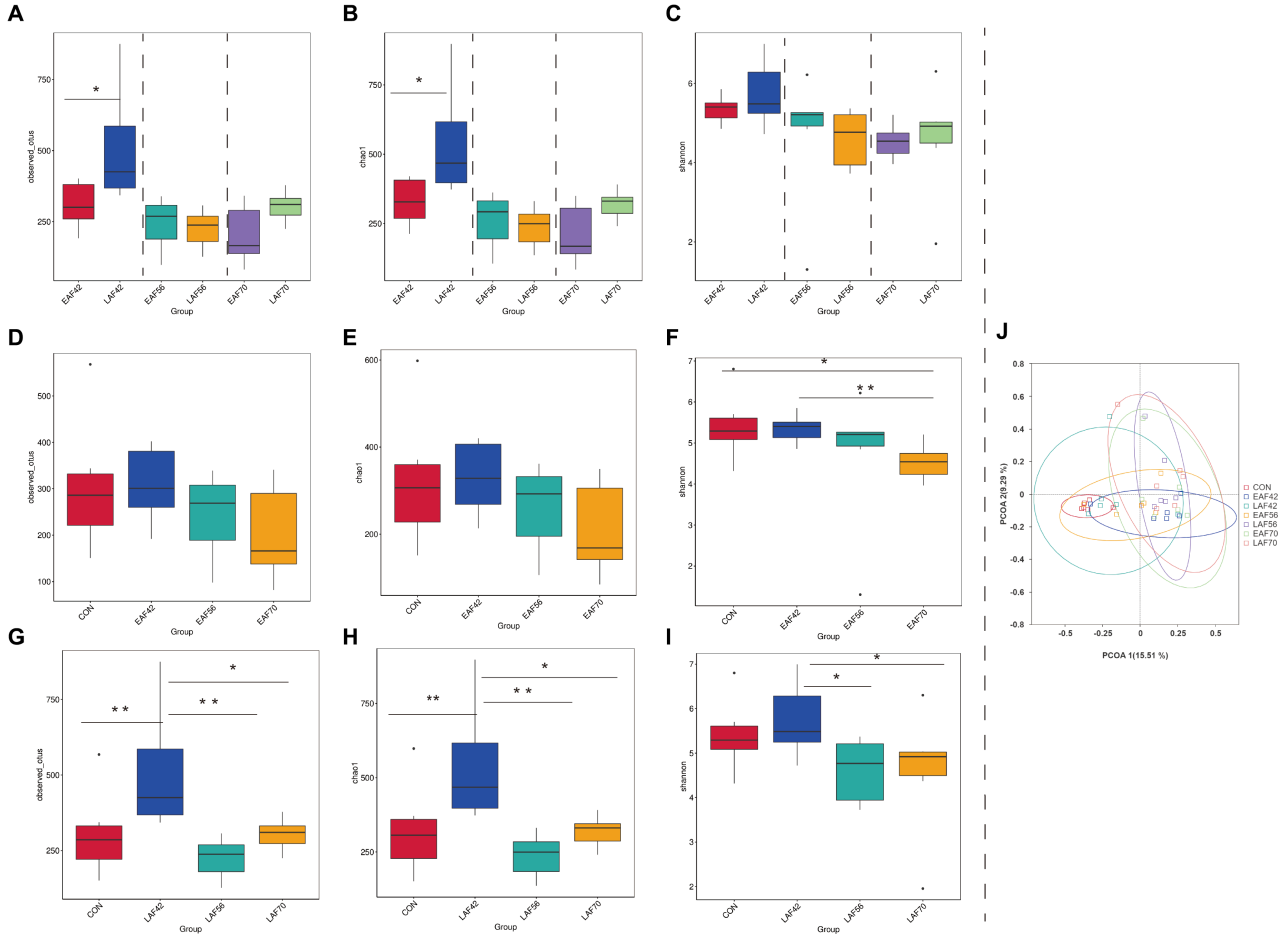
The effect of alfalfa supplementation timing on alpha and beta diversity of rumen fungi in pre-weaning lambs. Observed_OTUs **(A)**, Chao 1 **(B)**, and Shannon **(C)** indices in the rumen fungi between the EAF and LAF groups at 42, 56, and 70 days of age. Alpha diversity of the rumen fungi was analyzed using the Wilcox rank sum test. The rumen fungi diversities of the EAF group at 14, 42, 56, and 70 days of age **(D–F)**, and the alpha diversities of the LAF group at 14, 42, 56, and 70 days of age **(G–I)**. *p < 0.05, **p < 0.01, ***p < 0.001. The principal coordinate analysis (PCoA) was performed based on the Bray-Curtis dissimilarities metric **(J)**. EAF, lambs were fed alfalfa hay at 14 days of age as the early alfalfa feeding group; LAF, lambs were fed alfalfa hay at 42 days of age as the late alfalfa feeding group; CON, lambs were slaughtered at 14 days of age.

For the treatment effect, there was no significant difference in Observed_OTUs (Figure 6A), Chao 1 (Figure 6B), and Shannon (Figure 6C) indices between the EAF and LAF groups at 56 and 70 d of age. However, the Observed_OTUs (*p* < 0.05) (Figure 6A) and Chao 1 (*p* < 0.05) (Figure 6B) indices were higher in the LAF group than the EAF group at 42 d of age. For the age effect, the Shannon index was lower at 70 d of age than at 14 (*p* < 0.05) and 42 d (*p* < 0.01) of age (Figure 6F) in the EAF group. In the LAF group, the Observed_OTUs (Figure 6G) and Chao 1 indices (Figure 6H) were higher at 42 d of age compared to other time points (*p* < 0.05). The Shannon index was higher at 42 d than at 56 (*p* < 0.05) and 70 d (*p* < 0.05) of age (Figure 6I). In addition, PCoA analysis was performed based on the Bray-Curtis dissimilarities metric (Figure 6J) in the fungal community. The dissimilarities in the fungal communities between the EAF and LAF groups at 42, 56, and 70 d of age were not significant (ANOSIM, *R* = 0.07, 0.10, −0.11; *p* = 0.07, 0.06, 0.97). However, for the age effect, the dissimilarities in the fungal communities were evident between 42 d and 56 d (ANOSIM, *R* = 0.09, *p* = 0.02) or 56 d and 70 d (ANOSIM, *R* = 0.12, *p* = 0.01).

A total of 15 phyla were detected in all samples, among which Ascomycota and Basidiomycota were the most dominant phyla, accounting for over 68.13% of all reads (Figure 4C). At the genus level, a total of 532 distinct fungal genera were detected in all samples. The predominant fungal taxa in the collected samples included *Ascochyta, Naganishia, Wallemia, Alternaria*, and *Didymella*, accounting for over 33.89% of all reads (Figure 4D). To further understand how alfalfa supplementation timing affects the fungal community in pre-weaning lambs, we used MetaStat analysis to analyze the differences in the composition of rumen fungi between the EAF and LAF groups at 42, 56, and 70 d of age (Table 1). The abundance of *Naganishia* (*p* < 0.01), *Ascochyta* (*p* = 0.01), *Neosetophoma* (*p* = 0.01), *Kernia* (*p* = 0.01), and *Stagonosporopsis* (*p* = 0.02) were higher in the EAF than the LAF group at 42 d of age. However, the abundance of *Ascochyta* (*p* < 0.01), *Wallemia* (*p* = 0.03), *Aspergillus* (*p* = 0.04), and *Stagonosporopsis* (*p* = 0.01) were lower in the EAF than in the LAF group at 56 d of age. In addition, the abundance of *Aspergillus* was higher in the EAF group than LAF group at 70 d of age (*p* = 0.04). To further study the phylogenetic relationships of species at the genus level, the representative sequences of the top 50 genera were obtained by multi-sequence alignment, and the evolutionary tree of species at the genus level was drawn (Supplementary Figure S1). Thereinto, the genera *Ascochyta, Neosetophoma, Kernia*, and *Stagonosporopsis* belonged to phylum Ascomycota. The genera *Naganishia* and *Wallemia* belonged to the phylum Basidiomycota.

**Figure 7:**
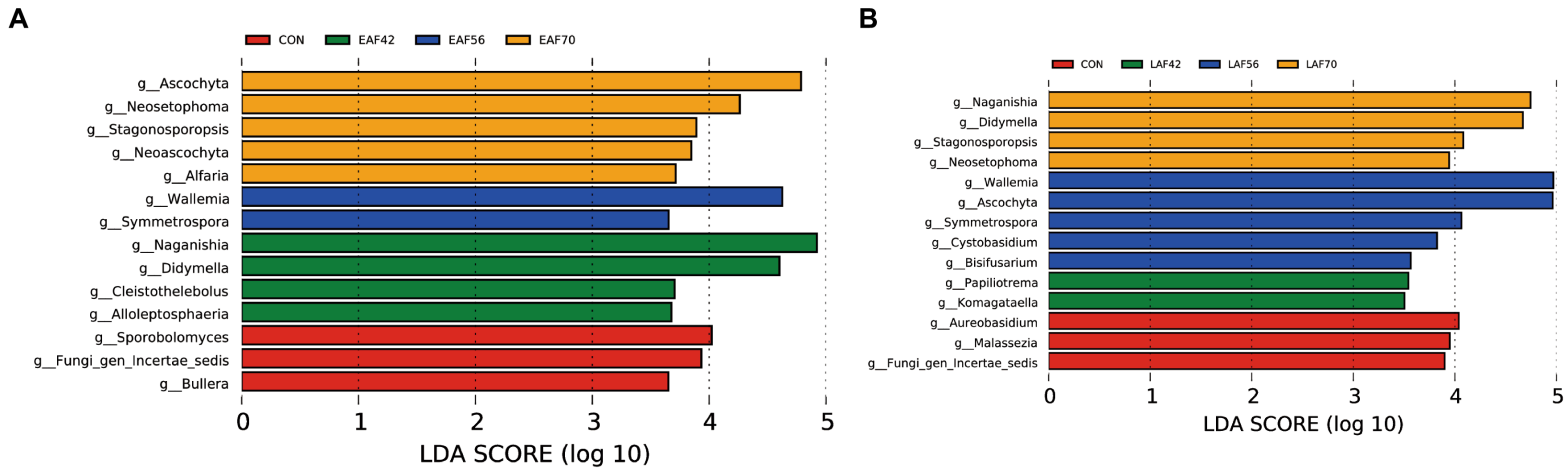
The rumen fungal biomarker (genus level) was identified by LEfSe analysis (LDA Score > 3.5) for each age in the EAF group **(A)** and the LAF group **(B)**. EAF, lambs were fed alfalfa hay at 14 days of age as the early alfalfa feeding group; LAF, lambs were fed alfalfa hay at 42 days of age as the late alfalfa feeding group; CON, lambs were slaughtered at 14 days of age.

For the age effect, we used LEfSe analyses to analyze the fungal biomarkers for both EAF (Figure 7A) and LAF (Figure 7B) groups changed with ages from 14 to 70 d of age. In the EAF group, *Ascochyta, Neosetophoma, Stagonosporopsis, Neoascochyta*, and *Alfaria* were higher at 70 d of age; *Wallemia* and *Symmetrospora* were enriched at 56 d of age; *Naganishia, Didymella, Cleistothelebolus*, and *Alloleptosphaeria* were abundant at 42 d of age; *Sporobolomyces, Fungi_gen_Incertae_sedis*, and *Bullera* were enriched at 14 d of age. In the LAF group, *Naganishia, Didymella, Stagonosporopsis*, and *Neosetophoma* were higher at 70 d of age; *Wallemia, Ascochyta, Symmetrospora, Cystobasidium*, and *Bisifusarium* were enriched at 56 d of age; *Paraphoma* and *Komagataella* were abundant at 42 d of age; *Aureobasidium, Malassezia*, and *Fungi_gen_Incertae_sedis* were enriched at 14 d of age.

### Correlation analysis of archaea and fungi in rumen samples

**Figure 8:**
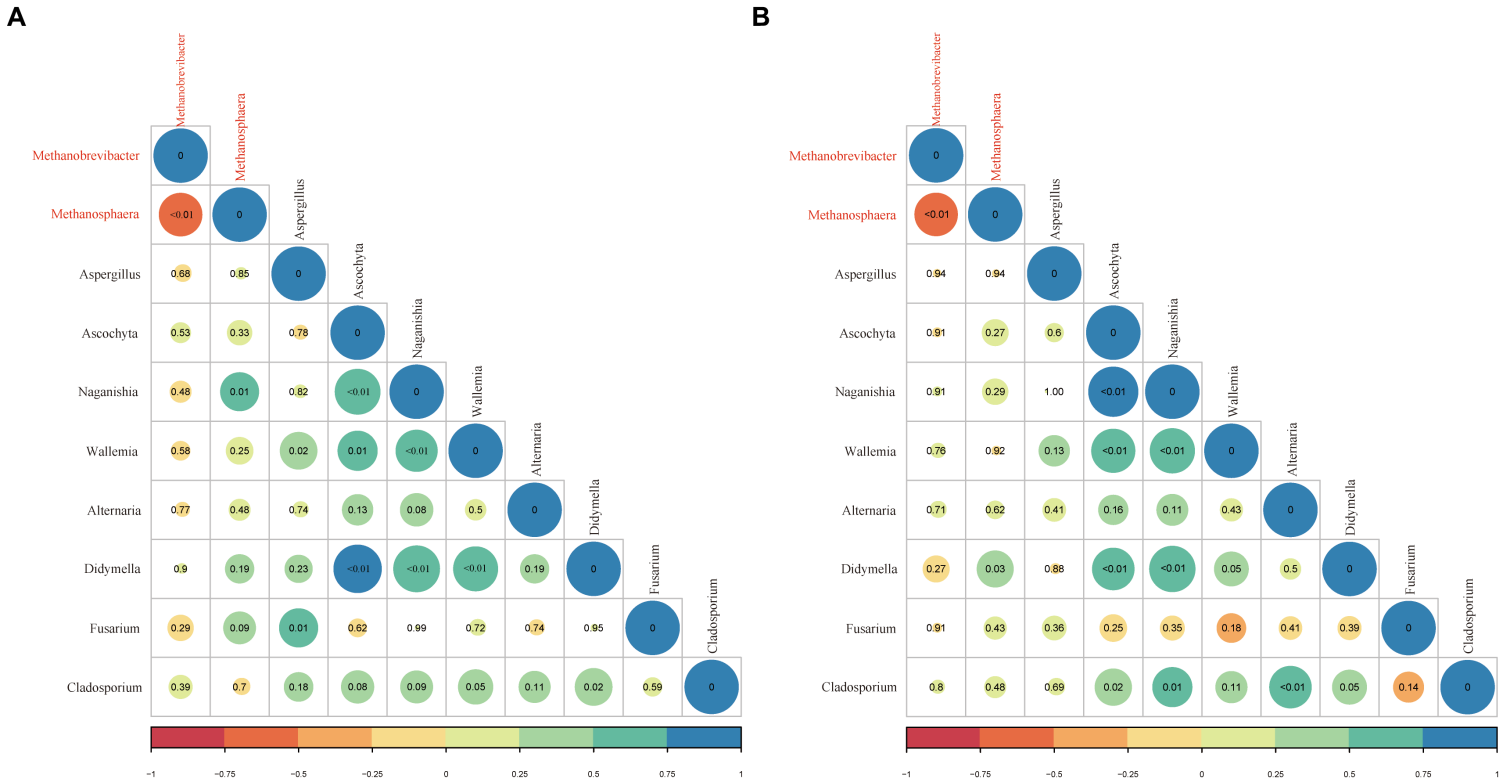
The correlation between archaeal (red) and fungal (black) taxa in the rumen samples of EAF **(A)** and LAF **(B)** groups. The correlation is shown by Spearman’s rank correlation, ranging from −1 to 1 and represented by color key red (perfect negative correlation) to blue (perfect positive correlation). The *p*-value is adjusted by the Benjamini-Hochberg method, and a p-value <0.05 indicates a significant correlation, with a trend considered at *p* < 0.10.

The correlation between the archaea and fungi in the rumen sample was assessed using the Spearmen rank correlation in the EAF and LAF groups (Figure 8). In the EAF lambs, the main associations between archaeal taxa included a negative correlation between *Methanobrevibacter* and *Methanosphaera* (*p* < 0.01). *Methanosphaera* was positively associated with *Naganishia* (*p* = 0.01). For associations between fungi, *Aspergillus* was positively associated with *Wallemina* (*p* = 0.02) and *Fusarium* (*p* = 0.01). *Ascochyta* was positively associated with *Naganishia* (*p* < 0.01), *Wallemina* (*p* = 0.01), and *Didymella* (*p* < 0.01). *Naganishia* displayed a positive association with *Wallemina* (*p* < 0.01) and *Didymella* (*p* < 0.01). *Wallemina* displayed a positive association with *Didymella* (*p* < 0.01) and tended to have a positive association with *Cladosporium* (*p* = 0.05). In addition, *Didymella* had a positive association with *Cladosporium* (*p* = 0.02). In the LAF lambs, the inter-intra archaeal taxa correlations were similar to those in the EAF group. For associations between archaea and fungi, *Methanosphaera* was positively associated with *Didymella* (*p* = 0.03). For associations between fungi, *Ascochyta* was positively associated with *Naganishia* (*p* < 0.01), *Wallemina* (*p* < 0.01), *Didymella* (*p* < 0.01), and *Cladosporium* (*p* = 0.02). *Naganishia* was positively associated with *Wallemina* (*p* < 0.01), *Didymella* (*p* < 0.01), and *Cladosporium* (*p* = 0.01). *Wallemina* tended to have a positive association with *Didymella* (*p* = 0.05). *Alternaria* was positively associated with *Cladosporium* (*p* < 0.01). In addition, *Didymella* tended to have a positive association with *Cladosporium* (*p* = 0.05).

## Discussion

### Performance

Supplementing hay during lactation can increase the intake of solid feed and help ruminants smoothly transition from liquid feed to solid feed during lactation. Research on calves (Daneshvar et al., 2015; Hosseini et al., 2015) and lambs (Norouzian et al., 2011; Yang et al., 2015) has shown that supplementing alfalfa hay before weaning will not result in a decrease in starter feed intake and body weight around weaning. In this study, we found that the total DMI of EAF lambs was 28.72% higher than that of LAF lambs from 28 to 42 d of age. During the period from 42 to 56 d of age, the intake of alfalfa hay in EAF lambs was 24.63% higher than that in LAF lambs. In addition, from 14 to 28 d of age, EAF lambs exhibited a 40.22% higher ADG than LAF lambs, while from 28 to 42 d of age, the ADG of EAF lambs was 32.22% greater. These results indicate that compared with alfalfa hay supplementation at 42 d of age, supplementing alfalfa hay to lambs at 14 d of age can promote their alfalfa hay intake, total DMI, and ADG. Xiao et al. (2023) reported that compared with calves feeding on hay in the second week after birth, calves starting to eat hay in the first week after birth would affect the nutritional digestibility and digestible nutrient intake. Hosseini et al. (2015) studied the effect of alfalfa hay supplementation time on the growth performance of calves and found that feeding alfalfa hay at 2 weeks of age can increase the feed intake and ADG of weaned calves. These results all suggest that the optimal time for ruminants to be supplemented with alfalfa hay before weaning is at 2 weeks of age. Castells et al. (2013) reported that the inclusion of alfalfa hay in the starter diet increased the body weight of calves, which was related to the filling effect in the gut, as the calves consumed more alfalfa hay. In addition, the normal development of the rumen muscle layer and rumen motility require sufficient physical stimulation (Beharka et al., 1998). Thus, alfalfa hay provision to young calves increased the thickness of the rumen wall muscle layer (Norouzian and Valizadeh, 2014). The development of the rumen muscle layer is another factor that increases the rumen volume and plays a significant role in the increase of rumen weight. The increase in rumen volume and weight is beneficial for the rumen to accommodate more feed. These results may partly explain why early supplementation of alfalfa hay can increase the total DMI and ADG of lambs. However, there were no significant differences in alfalfa hay intake, total DMI, and ADG between the EAF and LAF groups from 56 to 70 d of age in our study. The lambs in the LAF group were fed only with starter concentrate before 42 d of age. The starter concentrate contained a large amount of easily fermented carbohydrates (especially starch), which fermented rapidly and produced a large amount of volatile fatty acids, resulting in a decrease in the pH of rumen fluid (Suárez et al., 2006) and even ruminal acidosis, which ultimately inhibited feeding (Imani et al., 2017). On the contrary, when the lambs in the LAF group began to consume alfalfa hay at 42 d of age, it was beneficial to maintain a suitable rumen environment, accordingly increasing the alfalfa hay intake, total DMI and ADG. Therefore, there were no significant differences in alfalfa intake, total DMI and ADG between the EAF group and the LAF group from 56 to 70 d of age. Furthermore, studies have found that, due to their small particle size and the absence of abrasive components, starter concentrates have a weak ability to remove the stratum corneum of the rumen epithelium (Greenwood et al., 1997; Beiranvand et al., 2014). Therefore, feeding only starter concentrate can easily cause excessive thickening of the stratum corneum of young ruminants and the formation of adherent plaques of chyme on the rumen epithelium (Norouzian et al., 2011; Yang et al., 2015). Excessive thickening of the stratum corneum can reduce the absorption of VFA and blood flow in the rumen epithelium, and cause papillae degeneration and shedding (Beharka et al., 1998). The formation of adherent plaques of chyme on the rumen epithelium causes the aggregation of rumen papillae, reducing the absorption area of the rumen, which is not conducive to rumen health (Suárez et al., 2007). On the contrary, after lambs in the LAF group began to consume alfalfa hay at 42 d of age, due to the large particle size and high fiber content of alfalfa hay, it has a high roughness. Its continuous contact with the rumen epithelium can remove keratin or dead epithelial cells, making the rumen epithelium have a suitable degree of keratinization and integrity. Similar to our research findings, Castells et al. (2015) reported that early supplementation of forage could increase the total DMI and ADG of calves before weaning. However, after the calves in the starter concentrate-only group were supplemented with forage for two weeks, the differences in total DMI and ADG between the different treatment groups disappeared. Unfortunately, the researchers did not explain the biological mechanism behind this disappearance of the effect.

### Diversity of rumen archaea and fungi

The results of our study reveal the Observed_OTUs, Chao 1, and Shannon indices of archaea were higher at 14 d of age compared with 42, 56, and 70 d of age in the LAF group, with the main effect occurring at 42 d of age. At the same time, the PCoA analysis results showed that the structure of the rumen archaea at 14 d of age was distinct compared to the other ages, suggesting that the methanogens community changed significantly during the transition from a liquid to a solid diet. In addition, we found that the main methanogens (*Methanobrevibacter_sp_AbM4* and *Methanobrevibacter_smithii*) commonly observed in the mature rumen are colonized in the rumen of lambs at 14 d of age. These results suggested that the rumen archaea community of lambs was established before solid feeding (Guzman et al., 2015; Dias et al., 2017). Therefore, we propose that the most sensitive time to manipulate rumen methanogens through early intervention may be before solid feeding. Likewise, Yin et al. (2021) suggested that the period from birth to 20 d of age provides a unique window for manipulating the ruminal microbiota in lambs.

In the present study, the genera *Methanobrevibacter* (95.97 ± 6.30%) and *Methanosphaera* (2.94 ± 0.05%) were the dominant archaea in the EAF and LAF groups at different days of age. This is consistent with a previous study conducted by Li et al. (2023b) and Kumar et al. (2015). The genus *Methanobrevibacter* belongs to the group of hydrogenotrophic methanogens that produce CH_4_ through CO_2_ reduction. *Methanosphaera* is known to produce CH_4_ by reducing methanol using H_2_ (Shi et al., 2014). Wallace et al. (2015) showed that the relative abundance of *Methanosphaera* was higher in beef cattle with higher CH_4_ emissions than in beef cattle with lower CH_4_ emissions. Another study showed that *Methanosphaera* and *Methanomassiliicoccales* may have a greater share in overall CH_4_ production compared with *Methanobrevibacter* than previously thought (Söllinger et al., 2018). In the current study, the abundance of *Methanosphaera* tended to be higher in the EAF group compared to the LAF group at 42 and 56 d of age. These results suggested that early alfalfa supplementation may have increased methane emissions due to the increased abundance of *Methanosphaera*. The increase of *Methanosphaera* abundance in the EAF group at 42 and 56 d of age may be related to the pectin contained in alfalfa, and the main degradation product of pectin is methanol. *Methanosphaera* can use methanol to produce methane (Tapio et al., 2017). Peng et al. (2021) reported that the methanol-utilizing archaea *Methanosphaera stadtmanae* was enriched on alfalfa, which has the highest pectin content of the four substrates (alfalfa stems, bagasse, reed canary grass, and xylan). This may explain the reason for the increase in *Methanosphaera* abundance in the EAF group. Since the lambs in the LAF group only began to be fed alfalfa at 42 d of age, no changes in the abundance of *Methanosphaera* were observed at 56 d of age. This may be due to the short period of alfalfa intake by the lambs in the LAF group. The fact that there was no significant difference in the abundance of *Methanosphaera* between the EAF and LAF groups at 70 d of age proved this point. However, no matter how the *Methanosphaera* changes at 42 and 56 d of age, there was no significant difference between EAF and LAF groups at 70 d of age, indicating the effect of the early addition of alfalfa on the methanogen composition of the rumen may not persist in the long term and that the characteristics of the feed may play a much larger role. In addition, we explored the archaeal biomarkers (at the species level) at d 14, 42, 56, and 70 d of age in the EAF and LAF groups using LEfSe analysis. In the EAF group, *Methanobrevibacter_smithii* was abundant at 14 d of age. *Methanobrevibacter_smithii* (the members of the genus *Methanobrevibacter*) is associated with high CH_4_ production. Thus, this result supports the suggestion that manipulation of rumen methanogens through early intervention may be before solid feeding. However, we did not detect biomarkers of methanogens in the EAF group at 42, 56, and 70 d of age. In the LAF group, *Methanobrevibacter_smithii* was also abundant at 14 d of age. *Methanobrevibacter_sp_AbM4* was enriched at 56 d of age. No biomarkers have been identified for the archaea population at 42 and 70 d of age. Dong et al. (2019) reported that *Methanobrevibacter sp. strain AbM4* was the most abundant in the rumen of the early-weaned calves, and the butyrate was positively related to *Methanobrevibacter sp. strain AbM4*. Moreover, *Methanobrevibacter_sp_AbM4* produces CH_4_ from H_2_, CO_2_, and formate (Leahy et al., 2013). Feeding fat to beef cattle decreased the occurrence of *Methanobrevibacter_sp_AbM4* (Zhou et al., 2013). This may be related to the fact that high levels of dietary fat may inhibit protozoa. *Methanobrevibacter_sp_AbM4* has a positive relationship with two genera of protozoa (*Entodinium* and *Polyplastron*) (Zhou et al., 2013) and methanogens obtain H_2_ for methanogenesis through H_2_ transfer from protozoa (Tóthová et al., 2008).

At present, there have been few studies on the community structure of rumen fungi in pre-weaning lambs. Thus, we investigated the effect of alfalfa supplementation timing on the fungal community in the rumen of pre-weaning lambs at 14 to 70 d of age. It is generally assumed that anaerobic fungi could degrade the fiber by their efficient and extensive set of enzymes for the degradation of plant fiber (Solomon et al., 2016). Therefore, the inclusion of alfalfa in the diet generally increases the abundance of anaerobic fungi. Kumar et al. (2015) reported that the fungi were enriched in the rumen of cows fed a higher fiber diet. However, we found that the Observed_OTUs and Chao 1 indices were higher in the LAF group than in the EAF group at 42 d of age. This result suggested that early alfalfa hay feeding decreases the richness of the fungal community, but does not make a significant difference to the fungal diversity. This may be related to the imperfection of rumen physiology and functional development of degraded fiber before weaning. In addition, the Observed_OTUs and Chao 1 indices were significantly higher at 42 d of age compared with the other ages in the LAF group, but not in the EAF group. These results imply that the rumen fungal communities of pre-weaning lambs with early alfalfa supplementation were more stable than those with late alfalfa supplementation. The PCoA analysis showed that age has a significant effect on the community structure of the rumen fungi. In line with our results, Yin et al. (2022) reported that fungal populations in the rumen of lambs vary with age and the dynamic change of the fungal community can be divided into three phases: initial phase (0–10 d of age), transition phase (10–45 d of age), and a relatively stable phase (45–120 d of age).

In this study, the predominant fungal communities consisted of the phyla Ascomycota and Basidiomycota. Previous studies have reported that the main phyla in the rumen across all samples were Ascomycota, Basidiomycota, and Neocallimastigomycota (Kumar et al., 2015; Chaucheyras-Durand et al., 2016). However, the phyla Neocallimastigomycota average proportion < 0.1% in the present study. To understand the effect of alfalfa supplementation timing on the community structure of the rumen fungi in pre-weaning lambs. We used MetaStat analysis to analyze the differences in rumen fungal composition between the EAF and LAF groups at 42, 56, and 70 d of age. Specifically, the abundance of *Naganishia, Ascochyta, Neosetophoma, Kernia*, and *Stagonosporopsis* were higher in the EAF group than in the LAF group at 42 d of age. This suggests that early alfalfa supplementation can significantly increase the abundance of *Naganishia, Ascochyta, Neosetophoma, Kernia*, and *Stagonosporopsis*. The genera *Ascochyta, Neosetophoma, Kernia*, and *Stagonosporopsis* belonged to the phylum Ascomycota. Ascomycetes, as the largest group of microorganisms in the fungal kingdom, are mainly involved in the degradation of organic materials such as lignin and keratin during the digestion of nutrients (Beimforde et al., 2014), suggesting that early supplementation with alfalfa may promote fiber digestion in the diet. However, the abundance of *Ascochyta, Wallemia, Aspergillus*, and *Stagonosporopsis* was higher in the LAF group than in the EAF group at 56 d of age. Among them, the relative abundances of *Ascochyta* and *Stagonosporopsis* showed opposite trends at 42 and 56 d of age. These results suggested that the timing of alfalfa supplementation significantly affects the community structure of the rumen fungi. We also found that the abundance of *Aspergillus* was higher in the EAF group than LAF group even after being fed the same diet for four weeks, suggesting that the difference of *Aspergillus* between the EAF and LAF groups at 70 d of age is influenced by the timing of alfalfa supplementation, and the impact of this effect persists over a long period of time. In addition, we explored the fungal biomarkers at 14, 42, 56, and 70 d of age in the EAF and LAF groups using LEfSe analysis. We found that the timing of alfalfa supplementation significantly affected the establishment of rumen fungi. Specifically, the specific fungal biomarkers in the EAF group compared to the LAF group were *Ascochyta, Neoascochyta*, and *Alfaria* at 70 d of age, *Naganishia, Didymella, Cleistothelebolus*, and *Alloleptosphaeria* at 42 d of age, *Sporobolomyces* and *Bullera* at 14 d of age. However, compared to the EAF group, the fungal biomarkers specific to the LAF group at 70 d of age were *Naganishia* and *Didymella*, at 56 d of age were *Ascochyta, Cystobasidium*, and *Bisifusarium*, at 42 d of age were *Paraphoma* and *Komagataella*, at 14 d of age were *Aureobasidium* and *Malassezia*. This result suggests that the time of alfalfa supplementation has a significant effect on rumen microbial colonization, which also provides hope for the potential of early life programming interventions.

### Correlation analysis of anaerobic fungi and methanogens

In the rumen, there is a unique pattern of association between microorganisms. In the present study, the abundance of *Methanobrevibacter* was negatively correlated with *Methanosphaera* across the developmental stages of EAF and LAF lambs. *Methanobrevibacter* was the formation of CH_4_ through utilizing H_2_ + CO_2_ or formate, followed by a small percentage of *Methanosphaera* was the formation of CH_4_ through reduced methanol with H_2_ (Shi et al., 2014). Thus, the abundance of *Methanobrevibacter* and *Methanosphaera* showed a strongly negative association pattern, possibly due to competition for H_2_ within the rumen. In line with our results, Pitta et al. (2021) reported a negative correlation between *Methanosphaera* and *Methanobrevibacter*. There is a symbiotic relationship between rumen methanogens and fungi, and the co-culture of methanogens and fungi can increase the fiber degradation activity of fungi. When anaerobic rumen fungi are grown with methanogens significant increase in fungal biomass because of the removal of fermentation inhibitory intermediates (i.e., ethanol, formate, and lactate). Acetate, formate, H_2_, and CO_2_ were the major products of anaerobic fungi (Edwards et al., 2017; Li et al., 2019b). Many studies have now found that interaction between methanogens and anaerobic fungi, despite perturbations in dietary changes and aging (Dias et al., 2017; Pitta et al., 2021). Jin et al. (2011) reported *Methanobrevibacter spp.* associated with the *Piromyces, Anaeromyces*, and *Neocallimastix*. A novel methanogen species belonging to the uncultured archaea group (named Rumen Cluster C), subsequently called *Methanomassiliicoccus* (Borrel et al., 2014; Noel et al., 2016) was observed associated with anaerobic fungal cultures (Jin et al., 2014). We found that the abundance of *Methanosphaera* was positively correlated with *Naganishia* in the EAF group. However, in the LAF group, the abundance of *Methanosphaera* was positively correlated with *Didymella*. For the association between fungi, the abundance of *Ascochyta* was positively associated with *Naganishia, Wallemina*, and *Didymella* in the EAF group. However, *Ascochyta* is positively associated with *Cladosporium* in addition to *Naganishia, Wallemina*, and *Didymella* in the LAF group. The abundance of *Naganishia* was positively correlated with *Cladosporium* in the LAF group, but not in the EAF group. In addition, there was a trend towards positive correlation between *Wallemia* and *Cladosporium* in the EAF group, but not in the LAF group.These results suggested strong patterns of association both within and between archaea and fungi that can be influenced by alfalfa supplementation timing. Unfortunately, little is known about the function of these fungi in rumen digestion. Moreover, the intricate relationships both within and between methanogenic archaea and anaerobic fungi in the rumen ecosystem remain largely unexplored. Although our study provided new insights into the intra-microbial associations of the developing rumen, future studies should focus on the interactions between archaea and anaerobic fungi using both culture-independent and culture-dependent methods. In addition, it is necessary to investigate the rumen microbiome using metagenomic sequencing techniques to explore their functional role and interaction relation in lambs.

## Conclusion

In summary, initiating hay provision at 14 d of age, rather than at 42 d, could enhance total DMI and ADG among pre-weaning lambs. However, this effect disappears during the period from 56 to 70 d of age. The results of ruminal microbiota showed that supplementation with alfalfa hay began at 14 d of age increased the relative abundances of *Naganishia, Ascochyta, Neosetophoma, Kernia*, and *Stagonosporopsis* from fungi, suggesting that early alfalfa supplementation may promote dietary fiber digestion in lambs. For the age effect, the timing of alfalfa supplementation has a significant effect on rumen microbial colonization. In addition, the interaction both within and between archaea and fungi can be influenced by alfalfa supplementation timing. However, our understanding of the diversity and functional importance of methanogens and anaerobic rumen fungi associated with rumen remains incomplete.

## Data availability statement

The datasets presented in this study can be found in online repositories. The names of the repository/repositories and accession number(s) can be found below: NCBI − PRJNA1067551; PRJNA1067533.

## Ethics statement

The animal studies were approved by the experiment and animal procedures were done according to the “Laboratory Animal Guideline for Ethical Review of Animal Welfare” National Standard of the People’s Republic of China (GB/T 35892–2018). The studies were conducted in accordance with the local legislation and institutional requirements. Written informed consent was obtained from the owners for the participation of their animals in this study.

## Author contributions

KL: Investigation, Methodology, Software, Writing – original draft. HD: Formal analysis, Investigation, Writing – review & editing. WG: Data curation, Investigation, Writing – review & editing. MN: Data curation, Investigation, Writing – review & editing. RN: Conceptualization, Funding acquisition, Project administration, Supervision, Writing – review & editing.

## Footnotes

^1^ http://www.drive5.com/usearch/manual/uchime_algo.html

^2^ https://cloud.majorbio.com/page/tools/
