# Supplementary material for: Alfalfa supplementation timing changes the rumen archaeal and fungal community composition and colonization in pre-weaning lambs
Source: Front Microbiol. 2024 May 9;15:1380322. doi: 10.3389/fmicb.2024.1380322 (PMC11112515; doi:10.3389/fmicb.2024.1380322)
Supplement: Supplementary file 1 [file Table_1.DOCX]

Supplementary Material

Alfalfa Supplementation Timing Changes the Rumen Archaeal and Fungal Community Composition and Colonization in Pre-weaning Lambs

**Kenan Li, Haidong Du, Wenliang Guo, Meila Na, and Renhua Na***

College of Animal Science, Inner Mongolia Agricultural University, Hohhot, China

*** Corresponding author:**

Renhua Na, [narenhualaoshi@163.com](mailto:narenhualaoshi@163.com)

# 1. Supplementary Figures and Tables


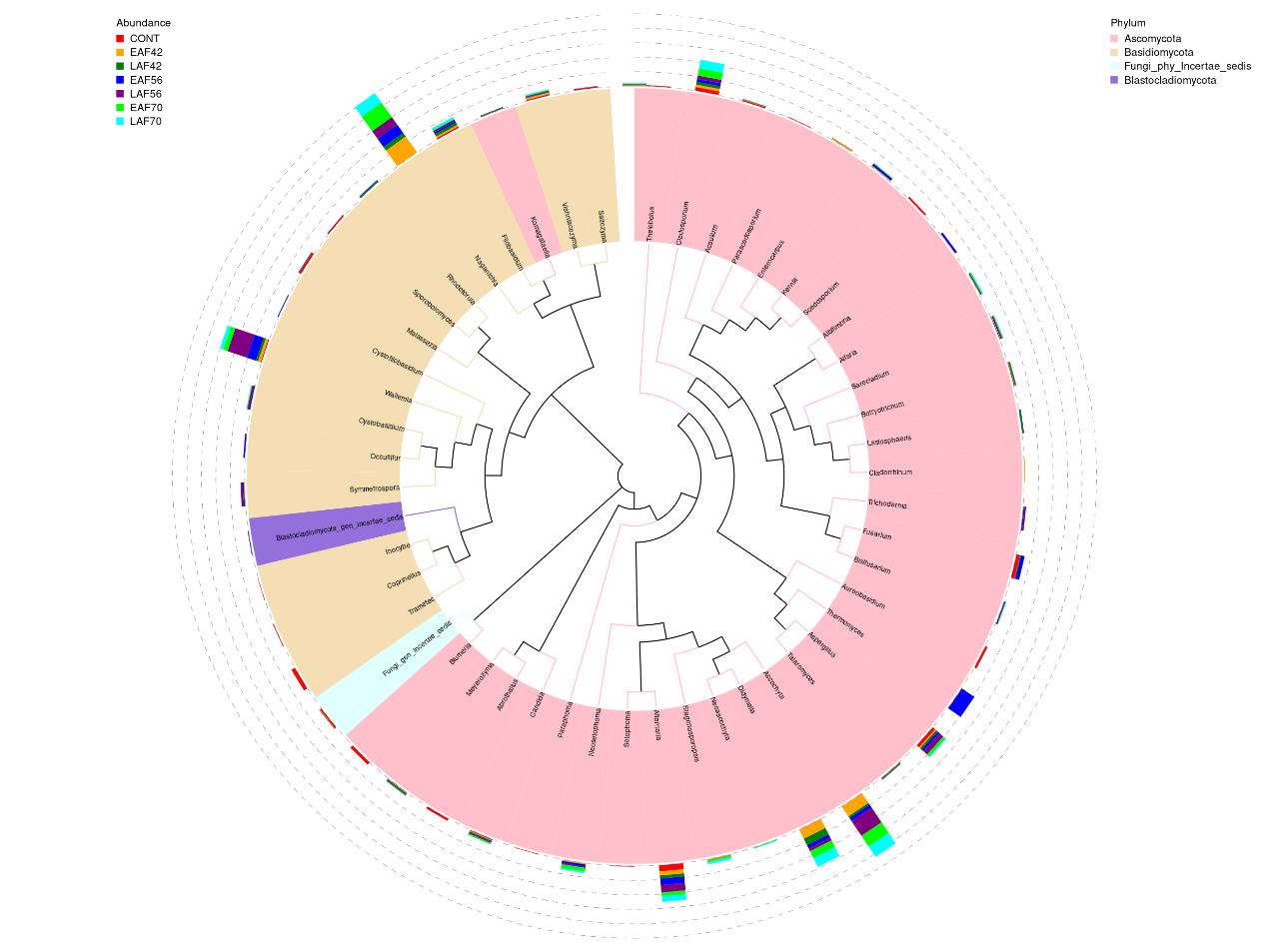


**Supplementary Figure 1.** Rumen fungal community (genus level) species evolutionary tree

Table S1. Nutrient components of milk replacer, starter pellets, and alfalfa hay (DM basis, %)

| **Items (%, unless otherwise stated)** | **Starter pellets** | **Milk replacer^3^** | **Alfalfa hay** |
| --- | --- | --- | --- |
| Ingredients | | | |
| Corn | 40.00 | - | - |
| DDGS | 3.00 | - | - |
| Soybean meal | 26.00 | - | - |
| Wheat bran | 13.00 | - | - |
| Corn germ meal | 6.00 | - | - |
| Soybean hulls | 4.00 | - | - |
| Extruded soybean | 4.00 | - | - |
| Limestone | 2.00 | - | - |
| CaHPO_4_ | 0.50 | - | - |
| NaCl | 0.50 | - | - |
| Premix^1^ | 1.00 | - | - |
| Total | 100.00 | - | - |
| Chemical composition | | | |
| DM | 89.70 | 94.45 | 86.63 |
| CP | 23.98 | 20.88 | 15.20 |
| EE | 4.86 | 11.08 | 2.23 |
| NDF | 22.46 | - | 55.96 |
| ADF | 8.10 | - | 43.07 |
| Ash | 7.48 | 2.59 | 7.09 |
| Ca | 0.98 | 1.05 | 1.35 |
| P | 0.72 | 0.68 | 0.16 |
| Metabolic energy (MJ/Kg)^2^ | 11.67 | 14.16 | 7.96 |

DDGS, distillers dried grains with solubles; DM, dry matter; CP, crude protein; EE, ether extract; NDF, neutral detergent fibers; ADF, acid detergent fiber; Ash, crude ash.

^1^Contained per kilogram of supplement: vitamin A, 800,000 IU; vitamin D3, 30,000 IU; vitamin E, 3000 mg; Cu, 0.8 g; Fe, 4 g; Mn, 4 g; Zn, 5 g; I, 70 mg; Se, 20 mg; Co, 40 mg.

^2^ Nutrient levels were all measured except the metabolic energy. The calculation method of metabolic energy refers to the method of Nutrient Requirements of Meat-type Sheep and Goats (NY/T 816-2021).

^3^ The milk replacer was stored in powder and consisted of whole milk powder, whey powder, protein concentrate, vitamin A (VA), VD3, VE, nicotinic acid, pantothenic acid, lysine, methionine, threonine, sodium chloride, copper, zinc manganese, and iron.
